# Mothers’ Self-Worth and Children’s Achievements: A Q Methodological Analysis of Perception Types

**DOI:** 10.3390/bs15050569

**Published:** 2025-04-23

**Authors:** Kyongmin Lee, Song Yi Lee, Sanghee Lee

**Affiliations:** Department of Counselling and Coaching, Dongguk University-Seoul, 30, Pildong-ro 1 gil, Jung-gu, Seoul 04620, Republic of Korea; km03012@naver.com (K.L.); songyilee@dongguk.edu.kr (S.Y.L.)

**Keywords:** child-based self-worth, psychological control, Q methodology

## Abstract

This study explores mothers’ subjective perceptions of self-worth in relation to their children’s achievements using Q methodology. A Q population was constructed based on previous studies and interviews. Forty Q sample statements were selected. Thirty-three mothers raising children were purposefully sampled to ensure representativeness in the P sample. The Q sorting procedure was conducted, followed by a principal component analysis using the Ken-Q Analysis Desktop Edition. The findings identified four distinct types of maternal self-worth perception: Type 1, Achievement-Independent, perceives self-worth independently from their children’s achievements and accepts their failures without attempting to control them. Type 2, Achievement-Dependent, considers children’s achievements crucial in their own life satisfaction and exhibits a strong tendency to control their children. Type 3, Ambivalent-Fusion, believes that children’s achievements do not directly affect their self-worth but shows an overprotective attitude to prevent their children’s failure. Type 4, Maternal-Obligation, perceives children’s achievements as a validation of their role as a mother and strongly internalizes the expectations and responsibilities associated with motherhood. This study categorizes mothers’ perceptions of self-worth and highlights the need for tailored support. The findings provide foundational data for the development of counseling services aimed at addressing mothers’ self-worth in relation to their children’s achievements.

## 1. Introduction

As the first significant figure a child encounters, mothers play a crucial role in the child’s development (Shim & Jung, 2018). While mothers strive to raise their children in an ideal manner (S. H. Lee, 2019), such expectations may lead to various role conflicts in parenting (E. J. Kim et al., 2014).

In Korean society, supporting children’s academic achievement is a core value of parenting. Consequently, Korean parents often initiate early education to ensure their children stay ahead in the highly competitive academic environment (Y. M. Han & Kwon, 2006). In a study conducted with mothers of school-aged children, 19.6% identified their child’s academic success as their greatest source of pride (Y. S. Park et al., 2002). This finding underscores the significant role of children’s academic achievements in the lives of Korean mothers.

Even within the broader social context of Korea, university admission is regarded as a crucial determinant of an individual’s social and economic status, making the competition for entrance exams extremely intense (Bank of Korea, 2024). Consequently, the College Scholastic Ability Test serves as a pivotal assessment that shapes students’ futures, leading parents to invest substantial money and effort into private education and early education to support their children’s academic success (KDI Economic Information Center, 2024).

The Korean educational culture, shaped by Confucian values, strongly emphasizes academic achievement and tends to regard children’s accomplishments as extensions of parental accomplishments (S. Han, 2008). In contrast, in Western individualistic cultures, parents and children are more likely to be perceived as independent entities, making such equivocations less pronounced (Kagitcibasi, 2005). Particularly in Korea, parents tend to regard their children’s academic achievements as personal accomplishments, which blurs the boundaries between parents and children (Choi & Yu, 2019).

Korea is also a collectivist society centered on kinship (S. I. Lee, 2010). In collectivist cultures, where individuals construct their self-concept within situational and relational contexts, mothers are more likely to incorporate their children into their own identity (S. Han, 2008). In such cultures, children’s achievements can significantly impact their mothers’ self-worth (Seol et al., 2015).

Accordingly, in kinship-based cultures that highly value academic achievement, mothers often perceive their self-worth as closely tied to the success of the children with whom they share the strongest familial interdependence (Markus & Kitayama, 1991), referred to as child-based self-worth (Ng et al., 2014). Accordingly, parents assess their worth based on their children’s academic performance (Eaton & Pomerantz, 2004). Mothers with high child-based self-worth tend to perceive themselves more positively when their children succeed; however, they may experience diminished self-worth and lower self-esteem when their children perform poorly or encounter failure (I. Y. Lee & Jung, 2019).

In Korean society, child-based self-worth is conceptualized as a psychological disposition that contributes to the excessive emphasis on academic achievement, particularly in the context of college entrance examinations (Seol et al., 2015). This construct extends beyond individual psychological tendencies, influencing children’s emotional and behavioral adjustment, academic motivation, and the overall quality of the parent–child relationship (M. H. Hong, 2022; Y. J. Kim, 2023; I. Y. Lee & Jung, 2019).

Furthermore, mothers with high child-based self-worth are more likely to exhibit heightened sensitivity to their children’s failures, which is associated with increased maternal anxiety and depression. These emotional difficulties, in turn, elevate parenting stress and the adoption of negative parenting behaviors (Choi & Yu, 2019; H. S. Park & Kim, 2023).

Collectively, these findings underscore the importance of further in-depth investigations into child-based self-worth within the cultural and psychological context of Korean parenting.

According to K. M. Lee and Lee (2024), a total of 22 research articles on child-based self-worth in Korea were published between 2014 and April 2024. Most of these studies analyzed the relationships between child-based self-worth and various psychological variables of parents and children. Notably, these studies relied exclusively on quantitative methods using self-report surveys.

Quantitative research has limitations in capturing the internal and subjective structure of perceptions such as self-worth (E. J. Kim, 2020). Q methodology is a research approach well suited for analyzing subjective perceptions, as it allows for the identification of comprehensive and multidimensional viewpoints (H. K. Kim, 2008). Furthermore, it can complement the explanatory limitations of traditional quantitative methods by uncovering individuals’ internal meaning structures and subjectivities rather than relying on surface-level response tendencies or mean-centered outcomes (Cross, 2005).

Accordingly, the present study deemed Q methodology appropriate for exploring mothers’ perceptions of self-worth.

In this study, relevant prior research was reviewed to collect a comprehensive set of concepts related to child-based self-worth for Q methodology. Among the variables examined in previous studies, psychological control was the most frequently investigated, with eleven studies reporting a positive correlation with child-based self-worth. Additionally, three studies on self-differentiation found a negative correlation with child-based self-worth, while two studies on motherhood ideology reported a positive correlation. A review of the literature revealed that psychological control, self-differentiation, and motherhood ideology emerged as key factors strongly associated with child-based self-worth (Ahn & Yoo, 2019; Y. A. Kim, 2021; J. H. Lee, 2017; B. H. Park & Baek, 2024; H. S. Park & Kim, 2023; Seol et al., 2015).

These constructs provide an important theoretical basis for understanding mothers’ subjective perceptions of child-based self-worth and their types (K. M. Lee & Lee, 2024).

The following presents theoretical definitions of these concepts and key findings from previous studies that demonstrate their associations with child-based self-worth.

Mothers with high child-based self-worth tend to equate their children’s success with their own success, resulting in a diminished sense of self-worth when their children fail (Eaton & Pomerantz, 2004). These mothers often prevent their children’s failure by developing a heightened fear of it and becoming excessively involved in their lives (Crocker & Wolfe, 2001). Such behaviors may contribute to psychologically controlling parenting (Grolnick et al., 2007).

Psychological control is a parenting practice in which parents regulate their children’s psychological domain by inducing guilt or withdrawing affection (Barber, 1996). Higher child-based self-worth in mothers has been associated with a tendency to interpret events involving their children negatively, which in turn leads to psychologically controlling parenting behaviors (H. S. Park & Kim, 2023). This form of parenting often creates tension in parent–child relationships, undermines children’s independence and autonomy, and reinforces their dependence. Over time, it can negatively impact children’s emotional well-being (Barber et al., 2005). Specifically, psychological control has been strongly associated with psychological issues such as anxiety and depression (A. R. Han et al., 2018; Jo & Kim, 2017), as well as aggression and oppositional behavior (J. J. Lee & Kim, 2021).

Another key variable associated with child-based self-worth is self-differentiation (S. H. Park, 2020). Self-differentiation is central in Bowen’s family systems theory, referring to an individual’s ability to separate cognition from emotion and maintain autonomy while remaining distinct from others (Skowron & Friedlander, 1998). Low self-differentiation in mothers has been found to increase both child-based self-worth and anxiety (Ahn & Yoo, 2019). Parents with high self-differentiation accept and respect their children as they are, fostering their autonomous development (Kerr & Bowen, 1988). In contrast, parents with low self-differentiation struggle to establish clear boundaries with their children and tend to equate their children’s achievements with their own achievements (Ahn & Yoo, 2019). Furthermore, they are more likely to engage in controlling behaviors to alleviate their own psychological distress (Sohn & Kim, 2020).

Motherhood ideology refers to socially constructed beliefs about women’s roles in child rearing. In Korea, this ideology was introduced through industrialization and Westernization and was integrated with Confucian patriarchal values, resulting in a culturally unique form that reflects traditional Korean norms (Yoon, 1996).

The traditional image of Korean mothers is represented by the ideal of the “wise mother and good wife” (*hyeonmo-yangcheo*), which emphasizes a woman’s role in raising successful children who contribute to society (J. K. Lee, 2004). Within this cultural framework, a mother’s competence is often evaluated based on her ability to care for her child and promote academic achievement, particularly through the child’s admission to a prestigious university (Shin, 2016).

In this context, motherhood ideology is closely aligned with Korea’s cultural emphasis on academic success. It serves as a significant psychosocial factor in understanding how mothers form their sense of self-worth based on their children’s achievements (B. H. Park & Baek, 2024). Furthermore, a greater internalization of motherhood ideology is linked to higher child-based self-worth, which ultimately increases parenting stress (J. H. Lee, 2017).

This study aims to classify the perception types of mothers’ self-worth based on their children’s academic achievements using Q methodology. In doing so, it seeks to provide a deeper understanding of the characteristics and underlying meanings associated with each type.

This study addresses the following research questions:

1. What are the distinct perception types of mothers’ child-based self-worth?
2. What are the comprehensive characteristics associated with each perception type?

## 2. Materials and Methods

### 2.1. Q Methodology Research Procedure

Q methodology was developed by William Stephenson, a British physicist and psychologist, in the 1930s to measure various aspects of human subjectivity (Stephenson, 1953). It integrates psychological, statistical, and philosophical principles to examine individuals’ intrinsic perspectives, such as values and beliefs, on a given topic (Brown, 1980). This methodology is employed to classify similar value systems or thought patterns among individuals in specific contexts and is widely used to investigate subjective human perceptions related to various topics (Herrington & Coogan, 2011).

In Q methodology, subjective perception refers to an internal framework through which individuals assign meaning to events or concepts. This framework encompasses personal beliefs, attitudes, values, and emotional responses and is shaped by one’s cultural and social background (Watts & Stenner, 2012).

Q methodology differs from R methodology in that it emphasizes human subjectivity rather than specific variables (B. J. Kim, 1999). It is used to systematically examine subjectivity through correlation analysis and factor analysis in a structured quantitative framework (Brown, 1980). To empirically investigate subjectivity, researchers analyze participants’ responses to structured statements and utilize a quantitative classification process to distinguish variations in perspectives. This approach integrates both quantitative and qualitative methods, enabling a more comprehensive examination of subjective viewpoints (Brown, 1997).

This methodology involves analyzing collected data based on individuals’ perceptions, allowing for the sorting of items while capturing unique responses and genuine perspectives from participants (Gil et al., 2020). Additionally, it is not designed to test pre-established hypotheses, but rather to enable researchers to discover new hypotheses regarding human subjectivity through participant responses (S. E. Ramlo, 2023). Furthermore, rather than passively responding to operational definitions set by the researcher, participants actively assign scores to statements, which allows them to extract previously unidentified subjective perceptions (Cross, 2005; S. Ramlo, 2016).

This study employed Q methodology to identify the perception types of mothers’ self-worth based on their children’s achievements and their views on controlling parenting. Additionally, the study analyzed the characteristics of each perception type and interpreted their underlying meanings. The research was conducted after obtaining approval from the Dongguk University Institutional Review Board (IRB) (IRB No. DUIRB2024-10-04). The research procedure consists of five stages, as follows: Q concourse (population) construction, Q sample selection, P sample selection, Q sorting by P sample, and data analysis. The detailed research procedure and content are summarized in Table 1.

#### 2.1.1. Q Concourse (Population) Organization

The Q concourse (Q population) refers to the comprehensive set of all items related to a research topic, forming an integrated whole (H. K. Kim, 2008). Constructing a Q concourse involves gathering all relevant items, which is a critical stage in research utilizing Q methodology (Jang & Whang, 2007). The components of the Q concourse can be expressed in various forms, including statements, photographs, images, and keywords. Importantly, the focus should not be on objective facts but rather on self-reference, reflecting the subjective projections of respondents (H. K. Kim, 2008).

In this study, the Q concourse encompasses all statements related to mothers’ self-worth based on their children’s achievements. A total of 244 statements were constructed through a review of the relevant literature, existing scales, and interviews with participants.

First, 219 statements were developed based on the Maternal Child-Based Worth scale, originally constructed by Eaton and Pomerantz (2004) and later translated and validated by Seol et al. (2015), along with relevant literature sources. The literature review included 22 studies on child-based self-worth, as reported in the scoping review by K. M. Lee and Lee (2024). These studies were selected based on a search conducted between 10 February 2024, and 10 May 2024, using the Research Information Sharing Service, DBpia (Nurimedia), and the Korean Studies Information Service System. The search terms included combinations of child-based, self-worth, and self-value.

Additionally, between 1 November 2024, and 10 November 2024, twenty-five statements were extracted through semi-structured interviews conducted with five mothers. The interview questions explored mothers’ thoughts and emotions related to their children, including how they feel when thinking about their child, what they wish for their child, and the moments they have felt proud of their child. Participants were also asked to reflect on what they consider most important for their child at this stage, how they feel as a mother when their child achieves something, and how they feel when their child fails to achieve something. Additionally, the interviews examined when they have felt a sense of self-worth in their daily lives, when they have felt valuable as a mother, and how they perceive differences in their self-worth when their child succeeds versus when they fail.

The interview participants consisted of five mothers raising children, and their demographic information is presented in Table 2.

#### 2.1.2. Q Sample Selection

The Q sample consists of statements selected from the Q concourse and serves as a critical factor in subsequent research findings. Thus, the selection of the Q sample is crucially important (H. K. Kim, 2008). At this stage, researchers must construct strong and distinct statements from the population, which represent the collective thoughts and opinions on the subject, ensuring that the selected statements do not overlap in meaning (Chang, 2021). A Q sample size that is too large may create difficulties in the later Q sorting process; therefore, selecting an appropriate number of statements is essential (H. S. Kim & Won, 2000).

Selecting the Q sample involves choosing statements that align with the research objectives and comprehensively represent opinions or perspectives related to the research questions (S. E. Kim, 2016). This process varies depending on the level of researcher involvement and the extent to which theoretical frameworks related to the research questions influence statement selection. However, an unstructured approach, in which statements from the Q concourse are categorized based on interviews and literature analyses under the researcher’s guidance, is more commonly utilized (Baek, 2015).

In this study, an unstructured approach was employed to comprehensively collect statements relevant to the research topic. An initial categorization was conducted based on the thematic similarity among the statements, followed by multiple rounds of re-categorization guided by the distinctiveness of meaning and the representativeness of each category. The statements were consequently organized into six categories that reflect maternal values, attitudes, and characteristics identified in previous studies on child-based self-worth (Y. O. Kim, 2022; J. H. Lee, 2017; I. Y. Lee & Jung, 2019; K. M. Lee & Lee, 2024; H. Y. Park & Kim, 2022; Seol et al., 2015). The categories are as follows: achievement-based worth, motherhood role, controlling parenting, independent worth, social evaluation, and fused identity.

The validity check during the Q sample selection process was conducted as follows:

In this study, for the selection of the Q sample, 244 statements from the Q concourse were organized in an Excel file, and 105 statements were eliminated through the removal of duplicates and the separation of statements containing multiple meanings.

Next, three doctoral students who took lectures on Q methodology reviewed the statements through repeated readings. They removed statements based on their relevance to the research topic, clarity of meaning, and the extent to which they reflected the conceptual dimensions identified in the study, resulting in the elimination of 94 statements.

A final expert review was conducted by two doctoral candidates with experience in Q methodology and two professors specializing in Q methodology and the relevant field. They excluded five statements based on criteria such as topic relevance and clarity of meaning, resulting in a final selection of 40 Q sample statements.

#### 2.1.3. P Sample Organization

The P sample refers to the study participants who participate in Q sorting. The size of the P sample, or the number of participants in Q sorting, varies depending on the research topic and objectives. If a study involves more than five factors and explores a complex topic, a larger sample is generally required. However, if the research aims to explore a topic rather than establish definitive conclusions, a smaller sample may be sufficient (H. K. Kim, 2008).

Since Q methodology focuses not on how certain characteristics differ among individuals but rather on the differences in significance within individuals, the P sample size is typically kept small to facilitate meaningful comparisons among factors and to fulfill the study’s discovery-oriented purpose (H. K. Kim, 2008; E. J. Kim, 2020). A sample size of 40 to 60 participants is commonly used (Stainton Rogers, 1995), and some researchers suggest maintaining a 1:1 ratio between the P sample size and the number of Q items (Watts & Stenner, 2005).

If the sample size is too large, responses may become skewed toward a single factor, making it difficult to distinguish specific characteristics and leading to statistical issues (E. J. Kim, 2020). Therefore, even with fewer than the commonly suggested 40 participants, meaningful research findings can still be obtained (H. S. Kim & Won, 2000). Participant selection is determined based on theoretical or practical considerations, with participants chosen for their theoretical relevance or availability (McKeown & Thomas, 2013). Additionally, participants are selected based on their perceived relevance to the research topic and their potential to provide meaningful insights (Jang & Whang, 2007).

In this study, participants were selected based on specific criteria, including interest in the presented topic, the ability to provide impartial opinions, or general curiosity about the subject (H. K. Kim, 1990). The P sample was recruited considering the research topic, specifically targeting mothers raising children. Research participation was advertised to this group, and a total of 33 mothers who agreed to participate were included in the study.

Participants were limited to mothers who had at least one year of parenting experience, with no fixed allocation based on the child’s developmental stage. Each participant was assigned an ID from P1 to P33, and data were collected on age and parenting duration.

Occupational categories were simplified into five groups to estimate the time mothers spend with their children: salaried employees, freelancers, self-employed individuals, full-time homemakers, and others. The details of the participants’ demographic characteristics are presented in Table 3.

#### 2.1.4. Q Sample Sorting

Q sorting involves P sample participants arranging the Q sample statements according to their subjective perspectives. Through this process, the results are modeled in a structured format (H. K. Kim, 2008). Two primary methods of Q sorting are free distribution and forced distribution. Free distribution allows participants to categorize statements freely, while forced distribution requires participants to classify statements according to a predefined format (Chang, 2021). Many studies employ forced distribution, utilizing a normal distribution framework for the sorting process (Brown, 1980; S. E. Kim, 2010), and the present study also adopted this approach.

During the sorting process, the researcher provided the final set of statements to the P sample and guided them through the Q sorting procedure. Prior to the sorting task, participants were informed about the study’s purpose and procedure, provided consent for participation, and completed a general characteristics survey. The Q sorting process was conducted between 27 November 2024 and 6 December 2024.

The researcher prepared a written guide to help the P sample easily understand the Q sorting procedure and provided detailed instructions throughout the process. Eighteen participants completed the Q sorting through face-to-face sessions, while the remaining fifteen received the materials in advance and participated via video call with real-time guidance. The sorting process took approximately 30 min, and as a token of appreciation, participants were given a coffee coupon upon completion.

**Figure 1:**
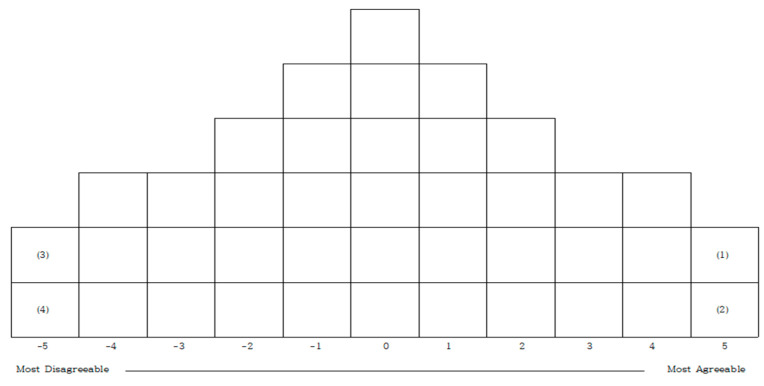
Q sorting distribution chart.

Participants were instructed to review the 40 Q sample statements and categorize them into three groups: agreement, disagreement, and neutrality, based on their subjective perceptions. After repeated reviews of the sorted statements, participants were then required to arrange them along an 11-point scale in a normal distribution format, as illustrated in Figure 1.

Following the completion of the sorting process, additional responses were collected to enhance the qualitative depth of the study and to enrich the dataset (Gallagher & Porock, 2010; E. K. Kim, 2020). Specifically, participants were asked to provide explanations for their selections regarding statements placed in the highest agreement (positions 1 and 2) and the lowest agreement (positions 3 and 4) category.

#### 2.1.5. Data Analysis

The study utilized Ken-Q Analysis Desktop Edition, version 2.0.1, for data analysis. To analyze the Q-sorted results from the 33 P sample participants, a scoring system was applied in which the most agreed-upon statements were assigned +5 points, while the most disagreed-upon statements received −5 points, with scores assigned sequentially.

Principal component factor analysis and varimax rotation were conducted to maximize the variance among factors and obtain optimal results (S. Ramlo, 2015). Based on eigenvalue reliability, a threshold of 1.00 was set, and four perception types were identified (J. S. Hong, 2023).

The Z-scores and Q-sorted values were used to extract statements that best represented each factor. Additionally, statements with high factor loadings, particularly those that participants most strongly agreed or disagreed with, were analyzed to identify and define the shared characteristics of each perception type.

## 3. Results

### 3.1. Analysis Results

Mothers’ self-worth based on their children’s achievements was categorized into four perception types. The eigenvalue for Type 1 was 11.55, followed by 3.21 for Type 2, 2.38 for Type 3, and 2.12 for Type 4, all exceeding the threshold of 1.00.

The explained variance accounted for 35% in Type 1, 10% in Type 2, 7% in Type 3, and 6% in Type 4, with a cumulative explained variance of 58% across the four types (see Table 4).

The correlations among the four perception types are outlined as follows. Correlations indicate the degree of similarity between types, with the values as follows: 0.0892 between Type 1 and Type 2, 0.5434 between Type 1 and Type 3, 0.5017 between Type 1 and Type 4, 0.1554 between Type 2 and Type 3, 0.179 between Type 2 and Type 4, and 0.5265 between Type 3 and Type 4.

In Q methodology, correlations are used to identify both commonalities and distinctions among types rather than assuming complete independence between factors, which is typically required in quantitative research. Therefore, the magnitude of the correlation values does not influence the identification of perception types (H. K. Kim, 2008). The analysis of the thirty-three P sample participants revealed four perception types. The distribution of participants across these types was as follows: 13 in Type 1, 5 in Type 2, 7 in Type 3, and 8 in Type 4. Details regarding factor loading, age, years of parenting, number and gender of children, and job category are presented in Table 3.

The Z-scores and Q-sorted values for the 40 Q statements are also presented in Table 3. The Z-score represents the standardized results of factor analysis and is used to assess the representativeness of each statement, providing insights into relative importance (Watts & Stenner, 2012). The Q-sorted value indicates the relative ranking of each statement based on participants’ agreement or disagreement levels in the Q sorting process (Brown, 1980). In this study, Z-scores and Q-sorted values were both utilized to analyze the characteristics and perception types of each group.

### 3.2. Perception Type Characteristics

In this study, four perception types regarding mothers’ self-worth and parenting attitudes based on their children’s achievements were identified. The four types are as follows: Type 1: Achievement-Independent, Type 2: Achievement-Dependent, Type 3: Ambivalent-Fusion, and Type 4: Maternal-Obligation. The characteristics of each type are as follows.

#### 3.2.1. Type 1: Achievement-Independence Type

The defining characteristic of Type 1 is that mothers view their self-worth independently of their child’s achievements. They believe that their child’s success does not influence how they evaluate themselves. The representative statements for this type, along with their corresponding Z-scores, are provided in Table 5.

The statements with the highest agreement include Q39 (“I believe my child’s failures are a natural part of their growth”) (z = 1.97), Q17 (“My child’s success or failure does not dictate the course of my life”) (z = 1.95), and Q29 (“Even if my child achieves great success, my sense of self-worth remains unchanged”) (z = 1.72). In contrast, the statements with the lowest agreement are Q26 (“When my child fails, I feel resentment toward them”) (z = −1.77) and Q36 (“When my child fails, the sense of satisfaction in life diminishes”) (z = −1.5).

P28 has the highest factor loading of 0.8657 and represents Type 1. The statement that received the highest agreement was Q17, and P28 mentioned the following:

“I believe that my child’s success or failure does not dictate the course of my life. My child’s life is their own, and my life is mine. I don’t feel the need to sacrifice myself to help my child achieve success. Achievements should be earned by the individual, and doing it for them is meaningless.”

P16 (0.8245) agreed most strongly with Q37 (“The value of my personal life does not fully align with the value of being a mother”) and stated the following:

“Before being a mother, I am an individual. While there may be similarities in the attitude I take toward my life as a mother and my life as an individual, I do not believe they align. I believe that being a mother requires a lot of strength, but…”

This type includes thirteen participants, and one participant, P10, has a factor loading of −0.8458, which stands in contrast to the typical characteristics of Type 1. This discrepancy warrants attention. The statements that P10 agreed most strongly with were Q22 (“I want to do whatever it takes to prevent my child from experiencing failure”) and Q26 (“When my child fails, I feel resentment toward them”). The statements with the lowest agreement were Q6 (“I am satisfied with my life, regardless of my child’s achievements or failures”) and Q17 (“My child’s success or failure does not dictate the course of my life”). P10 explained the following:

“Any parent would feel an intense desire to protect their child from failure, and I feel the same. The statement that I feel resentment when my child fails is true in my current situation. I am extremely resentful due to my child’s academic failure. My life is not satisfying because of my child’s failure. If my child attends a regional university, do I have to move there too? These depressing thoughts lead me to believe that my child is a determining factor in the direction of my life.”

Type 1 mothers perceive their self-worth independently of their child’s achievements and demonstrate an ability to accept the outcomes of their child’s successes and failures. Based on these findings, this type was interpreted as demonstrating lower dependence on children’s achievements for self-worth and a lower tendency toward controlling parenting patterns and was thus labeled as Achievement-Independent.

#### 3.2.2. Type 2: Achievement-Dependent Type

The defining characteristic of Type 2 is the great joy they derive from their child’s achievements. These individuals view their child’s success as one of the proudest moments of their lives and tend to see it as a reward for their own efforts. The statements that received the highest agreement in this type include Q40 (“My child’s achievements are the proudest moments of my life”) (z = 1.72), Q32 (“When my child achieves success, I feel that my efforts have not been in vain”) (z = 1.53), and Q30 (“I experience self-actualization through my child’s achievements”) (z = 1.45). The statements with the lowest agreement include Q1 (“A child’s achievements serve as a measure of my success”) (z = −2.16), Q12 (“My child’s achievements do not influence how others evaluate me”) (z = −1.42), and Q2 (“A child’s achievements reflect how well I have fulfilled my role as a mother”) (z = −1.39).

P5 (0.6557), who represents Type 2, agreed most strongly with Q15 (“When my child achieves great success, I experience a sense of stability”) and stated the following:

“When my child achieves success, I feel a sense of stability, and when they fail, I feel heartbroken. Logically, I understand that my child’s success is unrelated to my own, but when my child fails, I end up feeling a sense of failure in my heart, beyond just worrying.”

Another participant in Type 2, P9, agreed most strongly with Q20 (“I set high goals for my child to achieve success”) and explained the following:

“I don’t think a child’s failure is necessarily a mother’s problem. While genetics or environmental factors may play a role, I believe a child’s effort and willpower are what truly matter. Since I think success is strongly related to setting goals, I tend to set high goals for my child.”

Type 2 mothers derive great satisfaction from their children’s achievements and, through these outcomes, experience a sense of self-worth as mothers. This type tends to try to control the outcomes of their child’s successes and failures by being deeply involved in their children’s education. Based on these characteristics, this type was interpreted as exhibiting a higher child-based self-worth and a higher psychological control. Accordingly, it was labeled Achievement-Dependent.

#### 3.2.3. Type 3: Ambivalent-Fusion Type

Type 3 mothers agree that their self-worth does not change even if their children achieve great success, but they also believe that their children’s achievements are a critical factor in their mental well-being. Furthermore, they are willing to invest their resources freely to ensure their children’s success. When it comes to failure, they either want to prevent their children from experiencing it or feel as if they would like to take on the failure themselves. The statements that received the highest agreement in this type include Q29 (“Even if my child achieves great success, my sense of self-worth remains unchanged”) (z = 1.87), Q5 (“I spare no time or money in investing for my child’s success”) (z = 1.56), Q11 (“My child’s achievements are an important factor in my mental health”) (z = 1.27), Q22 (“I want to do whatever it takes to prevent my child from experiencing failure”) (z = 1.16), and Q25 (“I sometimes wish I could take on my child’s failures for them”) (z = 1.12). In contrast, the statements with the lowest level of agreement include Q9 (“When my child fails, I tend to discipline them harshly”) (z = −2.12), Q26 (“When my child fails, I feel resentment toward them”) (z = −2.05), and Q16 (“My child’s achievements define who I am as a person”) (z = −1.36).

P27, with a factor loading of 0.8339, represents Type 3. P27 agreed most strongly with Q29 and stated the following:

“I don’t think my child’s success is directly connected to my success, but I want to raise my child in a way that benefits them. I believe that in order for my child to develop in the right, comfortable, and easy way, parents must invest their best efforts and make sacrifices. Since my child still has much to learn, I believe learning is necessary.”

Another participant in Type 3, P20 (0.7026), agreed most strongly with Q5 and stated the following:

“I don’t think my child’s achievements define my life. My goal is to remain unaffected by my child’s achievements. If parents are shaken by their child’s success or failure, it will make the child anxious as they move to the next stage. I believe that as a parent, I must stay grounded. There are things I regret not doing at the right time while raising my child. Just like height, if you miss the right timing, no amount of money or time can bring it back. Although the time and money spent may not feel rewarding, I believe that living with regret for the rest of my life would be much harder, which is why I invest my time and money freely for my child’s success.”

P2 (0.6917) agreed most strongly with Q28 and Q29 and stated the following:

“Raising my child is one of my many abilities. I feel a sense of achievement when I accomplish something in areas other than parenting. When my child succeeds greatly, it feels like a reward. However, I don’t think it will drastically change my life or my value. If my ability to educate my child is recognized, and I have the opportunity to open a tutoring center or demonstrate my capabilities socially, then I believe my value could increase.”

Type 3 mothers believe that the outcomes of their children’s achievements do not influence their own sense of self-worth. However, they put considerable effort into attempting to control whether their child succeeds. Based on these characteristics, this type was interpreted as exhibiting a moderate child-based self-worth and a higher tendency toward controlling parenting patterns and was therefore labeled Ambivalent-Fusion.

#### 3.2.4. Type 4: Maternal-Obligation Type

Type 4 is characterized by mothers who show an accepting attitude toward their children’s failures but feel a sense of relief when their children succeed. They perceive their children’s success as a reward for their own life and believe that a mother’s sacrifice is necessary for their children’s achievements. They see their children’s success as confirmation that they have fulfilled their motherly role. The statements that received the highest agreement in this type include Q27 (“When my child achieves success, I feel a sense of relief”) (z = 1.54), Q23 (“When my child succeeds, I feel it is a reward for my life”) (z = 1.48), Q18 (“A mother’s sacrifice is necessary for her child’s success”) (z = 1.24), and Q2 (“A child’s achievements reflect how well I have fulfilled my role as a mother”) (z = 1.11). The statements with the lowest agreement are Q38 (“Academic success is the top priority in my child’s education”) (z = −1.64), Q12 (“My child’s achievements do not influence how others evaluate me”) (z = −1.24), and Q6 (“I am satisfied with my life, regardless of my child’s achievements or failures”) (z = −1.11).

P30, a participant who demonstrates the characteristics of Type 4 (0.6374), agreed most strongly with Q2 and Q23 and stated the following:

“I believe that as a mother, I need to provide mental support and help my child grow in order for them to achieve success. Achieving success requires effort from me as a mother, and that is why I feel a sense of pride in their accomplishments.”

Another participant in Type 4, P1 (0.614), stated the following:

“It seems that society places the responsibility for my child’s achievements and failures on me. I feel that this societal expectation is making my life as a mother increasingly difficult. While blaming others is pointless, I strongly dislike the social atmosphere that assigns this responsibility to mothers.”

P29 (0.6175) explains the characteristics of Type 4 with the following statement:

“I believe that raising a child and ensuring their proper growth is the role of a mother. I think there is no parental role without sacrifice.”

Type 4 mothers believe that it is the mother’s role to ensure their children’s success. While they recognize that they cannot control their children’s achievements, they believe that a mother’s sacrifice is necessary for her children’s success. Based on these characteristics, this type was interpreted as exhibiting higher dependence on children’s achievements for self-worth and a moderate tendency toward controlling parenting patterns and was therefore labeled Maternal-Obligation.

## 4. Discussion

This study explores the perception types of mothers’ self-worth based on their children’s achievements and identifies four types through Q factor analysis.

Type 1, Achievement-Independent, mothers view their children as independent entities, separate from their mothers. These mothers derive their self-worth from their own goals and outcomes rather than their children’s achievements. They recognize that their values and standards of evaluation are not applicable to their children and believe that children should experience and grow independently. These mothers do not perceive their children’s failures as their own. Instead, they perceive their children’s failures as part of the growth process and actively support their children’s autonomy. Consequently, they have little inclination to control their children.

Mothers in this type tended to be younger and more likely to be employed compared to those in other types. This pattern aligns with the characteristics of women who redefine the meanings of work, parenting, and family and pursue self-actualization through their careers rather than passively internalizing traditional notions of motherhood (S. Kim, 2022).

This type shows a trend consistent with the findings of research suggesting that the higher the child-based self-worth, the higher the psychological control exhibited by parents (Ching, 2023). When parents seek to validate their self-worth through their children’s achievements, they tend to engage in evaluating and controlling their children’s success (Wuyts et al., 2015). However, in the case of Type 1, self-worth is experienced separately from the children’s achievements, and these parents are more accepting of their children’s failures, showing lower tendencies toward controlling parenting.

Respondents in this type exhibit a relatively stable self-worth pattern, with high self-differentiation, where their identity as a parent is separate from that of their children. When the level of self-differentiation is high, individuals tend to maintain independence and objectivity, acting according to their beliefs (Ha, 2007; B. H. Kim & Song, 2014), which aligns with Type 1. Additionally, research suggests that mothers with higher self-differentiation tend to grant autonomy to their children and exhibit lower tendencies toward controlling parenting (Glebova, 2003).

In summary, Type 1 can be interpreted as exhibiting a low influence of the child’s achievements on the mother’s self-worth and a lower tendency toward controlling parenting.

Type 2, Achievement-Dependent mothers believe that their children’s achievements are a significant factor in their own life satisfaction. For these mothers, their children’s success holds great meaning in their lives, and through it, they experience the reward of their efforts and a sense of self-actualization.

This type includes five respondents, and examining their parenting duration provides important insights. Four of them had between 15 and 20 years of experience, while one had between 10 and 15 years. All of them were mothers of school-age children. In Korea, parents of school-age children are expected to take on various roles to improve their children’s academic performance during the intense college entrance preparation period (E. J. Kim, 2020). Furthermore, mothers of school-age children experience more stress related to their parental roles compared to mothers of younger children, with stress related to academic achievement and private education being particularly exacerbated (Kang, 2003).

The characteristics of Achievement-Dependent mothers align with the typical image of parents in Korean society. Research analyzing differences in educational fervor across countries indicates that Korean parents tend to realize their aspirations through their children’s education and view education as a tool for elevating social status (J. G. Lee, 2003). This tendency leads parents to focus excessively on education, creating normative expectations that children will become more educated than their parents. It also intensifies the obsession with their children’s achievements and strengthens the desire to control their children’s failures (J. H. Lee, 2017). In fact, in Korean society, a child’s university entrance is sometimes seen as a measure of the mother’s role fulfillment (J. H. Lee & Bae, 2013).

Similarly, Ng et al. (2013), who analyzed the relationship between mothers’ beliefs about their children’s learning and child-based worth in Hong Kong and the United States, found that mothers in Hong Kong tended to perceive their children’s academic achievement as controllable and evaluated their own self-worth based on it. In contrast, American mothers viewed learning as more difficult to control and were less likely to associate their self-worth with their children’s achievements. These findings suggest that mothers’ attitudes toward children’s academic success and self-concept may be shaped differently depending on mothers’ cultural backgrounds. Achievement-Dependent mothers exhibit attitudes more similar to those of Hong Kong mothers than to those of American mothers, demonstrating a strong tendency to link their self-worth with their children’s achievements.

Thus, the Achievement-Dependent type is more likely to exhibit higher child-based self-worth and a more controlling parenting style compared to other types. This finding aligns with previous findings by Ng et al. (2014), which indicate that higher child-based self-worth is associated with increased parental psychological control. Additionally, it is consistent with the longitudinal study by Wuyts et al. (2015), which demonstrated that Achievement-Dependent psychological control varies depending on the level of child-based self-worth.

Consequently, Achievement-Dependent mothers derive the greatest joy from their children’s achievements and are more likely to adopt a controlling parenting style to prevent failure.

The Ambivalent-Fusion type includes seven respondents, and its core characteristic can be summarized by the word “ambivalent”. These mothers perceive their self-worth as unchanged and report being satisfied with their lives regardless of their children’s achievements. However, they simultaneously consider their children’s success a crucial factor in their own mental well-being. Additionally, they exhibit a strong tendency to avoid their children’s failures to the extent that they would prefer to experience them on their behalf, willingly investing significant amounts of time and financial resources to prevent such setbacks.

These mothers exhibit low self-differentiation. Research findings (J. Y. Park, 2024) indicate that parents with low self-differentiation struggle to establish clear boundaries between themselves and their children, often attempting to solve their children’s problems on their behalf or becoming excessively involved in their children’s lives. This finding aligns with the characteristics of the Ambivalent-Fusion type.

Research on self-differentiation suggests that parents with low self-differentiation tend to form enmeshed relationships with their children and are more likely to evaluate their self-worth based on their children’s achievements (Ahn & Yoo, 2019; Y. A. Kim, 2021; S. H. Park, 2020; H. Y. Park & Kim, 2022). Additionally, individuals with low self-differentiation struggle to distinguish themselves from others and fail to maintain independence within close relationships (S. K. Lee et al., 2012). However, Ambivalent-Fusion mothers exhibit an ambivalent characteristic in that, despite maintaining a fused relationship with their children, they perceive their self-worth as independent of their children’s achievements.

Parents with low self-differentiation tend to prioritize their own needs over their children’s growth and development, often exhibiting overprotective behaviors to fulfill these needs (C. O. Park & Cho, 2011). Additionally, they perceive their children’s problems as their own and respond in a fused manner (Peleg, 2005), demonstrating a tendency to control their children as a means of alleviating their own psychological distress, particularly anxiety (Sohn & Kim, 2020). These findings align with the characteristics of the Ambivalent-Fusion type.

Considering these results, Ambivalent-Fusion mothers perceive their self-worth as independent of their children’s achievements. However, they are likely to engage in overprotective and controlling parenting behaviors.

The Maternal-Obligation type includes eight respondents. These individuals do not attempt to control their children’s failures or achievements and tend to perceive failure as a necessary component of their children’s growth. However, their children’s success provides them with psychological comfort, which they regard as a reward. Additionally, they believe that sacrifices are necessary for their children’s success and perceive the outcomes as a measure of the fulfillment of their maternal role.

Mothers of this type perceive their children as central to their lives and devote a significant amount of emotional energy to them, reflecting the traditional image of Korean mothers (S. Y. Kim & Jung, 2013). Despite the increasing social and economic participation of women in modern society, childcare and education are still largely regarded as a mother’s responsibility, a notion that persists within the concept of “motherhood” (Shin, 2016). The idealized image of a “good mother” influences women’s consciousness and behaviors, shaping their personal beliefs accordingly (J. H. Lee & Bae, 2013).

Perceiving the sacrifice of one’s rights and desires for child rearing as a natural duty reflects a high acceptance of maternal ideology, indicating that these mothers consider their maternal role a crucial aspect of their lives (J. H. Lee, 2017). These mothers invest significant amounts of time and effort into their children, and, as a result, their children’s achievements profoundly impact their self-worth (Grolnick et al., 2007).

Accordingly, these mothers tend to perceive their children’s achievements as having a relatively strong impact on their self-worth. However, given their attitude toward their children’s failures, their tendency toward controlling parenting can be interpreted as moderate.

The distinct characteristics of child-based self-worth and tendencies toward controlling parenting suggest that a differentiated counseling approach is necessary for each type when addressing parent–child conflicts.

Achievement-Independent mothers, characterized by low child-based self-worth and a low tendency toward controlling parenting, tend to respect their children’s autonomy and maintain a certain distance from them. However, it is important for these mothers to assess how well they are fulfilling their children’s emotional needs and how attachment has been formed. Inadequate emotional support may lead to the development of insecure attachments (Ainsworth et al., 1978), and, if parental involvement is excessively low, children may instead experience psychological distress (Soenens & Vansteenkiste, 2010).

Parenting styles that emphasize individual independence and self-reliance are common in Western cultures. However, in Korean society, such approaches may be perceived as creating emotional distance, while parental control is often interpreted as an expression of love (I. Y. Lee, 2022). Considering these cultural factors, therapeutic interventions such as emotionally focused therapy (Johnson, 2004), which facilitates emotional expression and supports the formation of secure attachment; parent–child interaction therapy (Eyberg et al., 2008), which fosters positive parent–child interactions; and theraplay (Booth & Jernberg, 2010), which enhances parental sensitivity and supports children’s emotional regulation, may be effective.

Achievement-Dependent mothers, characterized by high child-based self-worth and high tendencies toward controlling parenting, exhibit a strong inclination to seek personal fulfillment through their children’s achievements. Consequently, conflicts between parents and children may frequently arise. In particular, mothers’ self-worth fluctuates significantly depending on their children’s level of achievement, which can lead to negative emotions toward their children and increased psychological control. Ultimately, this may contribute to the mothers’ own experiences of depression (Choi & Yu, 2019).

Therefore, interventions based on cognitive behavioral therapy may be effective in assessing mothers’ psychological states and modifying irrational beliefs about success. Through this process, mothers can develop a greater awareness of the negative emotions arising in their relationships with their children, which may help reduce their tendency toward controlling parenting (Tsang & Lam, 2023). Additionally, acceptance and commitment therapy can be utilized to help mothers recognize and accept negative emotions while employing strategies to manage stress (Hayes et al., 2006).

Ambivalent-Fusion mothers, characterized by low child-based self-worth and high tendencies toward controlling parenting, tend to form enmeshed relationships with their children, struggling to establish clear psychological boundaries. Therefore, addressing conflicts in the parent–child relationship requires regulating ambivalent attitudes and improving low self-differentiation.

Recognizing the enmeshed relationship with their children and maintaining a healthy psychological distance through decentering is crucial for establishing clear boundaries. To achieve this, emotionally focused family therapy can effectively help parents explore their emotions and restructure maladaptive emotional patterns (Mikulincer & Shaver, 2016). Additionally, dialectical behavior therapy can help mothers integrate ambivalent emotions, enhance emotional regulation, and develop healthy interpersonal skills (Linehan et al., 2006).

Maternal-Obligation mothers, characterized by high child-based self-worth and moderate controlling parenting, hold a strong awareness of maternal roles and obligations, reflecting the influence of intensive mothering ideology. However, given the cultural characteristics of Korean society, modifying such beliefs may be difficult.

Therefore, narrative therapy can effectively help mothers reconstruct their life stories in a new and meaningful way (White & Epston, 1990). Additionally, group programs incorporating mindfulness-based interventions (K. E. Kim & Kim, 2015) and schema therapy (Kwon & Choi, 2021) for mothers with similar experiences can help reduce psychological stress and facilitate positive change.

Research on child-based self-worth in South Korea began around 2014 and has gradually increased over time. However, most existing studies have focused on measuring the degree of child-based self-worth using relevant scales and examining its mediating or moderating effects on related psychological variables (K. M. Lee & Lee, 2024). Although E. J. Kim (2020) conducted a Q methodological study exploring parents’ subjective perceptions of the value of their children and identified distinct perception types based on developmental stages, no studies have applied Q methodology specifically to the topic of child-based self-worth.

In E. J. Kim’s (2020) study, parents’ perceptions of their children’s value varied according to the child’s developmental stage. For parents of infants and toddlers, three types emerged: those who felt satisfaction simply from their child’s existence, those who emphasized devotion and patience, and those who valued their child as a contributor to social worth. Among parents of school-aged children, the types included those who viewed their children as independent beings, those who found joy in sharing the growth process, and those who sought growth through emotional fusion with their children. For parents of adolescents, the types identified were those who held positive expectations for their child’s growth, those who endured dissatisfaction with patience, and those who internalized their own hopes and aspirations within their children. These findings suggest that parents’ perceptions of their children’s value are influenced by their psychological characteristics as well as by the developmental stage of the child.

This study provided a comprehensive understanding of child-based self-worth, a distinctive characteristic of Korean mothers, and contributed to interpreting parenting attitudes that may lead to conflicts in the parent–child relationship. Furthermore, this study can serve as a practical foundation for the development and implementation of future psychotherapy and counseling programs tailored to the characteristics of each perception type.

## 5. Conclusions

**Figure 2:**
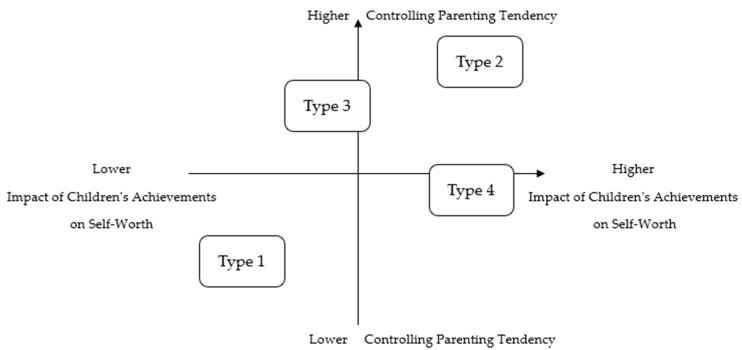
Parenting typology by types.

This study analyzed the impact of children’s achievements on mothers’ self-worth and classified them into four distinct types. Additionally, based on the findings, the study visualized the relationship between mothers’ self-worth and tendencies toward controlling parenting in a structured model (Figure 2).

Achievement-Independent mothers perceived their children’s achievements and their own self-worth as separate and demonstrated an accepting attitude toward their children’s failures without attempting to control them. Achievement-Dependent mothers exhibited a strong tendency to control their children, as their children’s achievements directly impacted their self-worth. Ambivalent-Fusion mothers perceived their self-worth as not significantly influenced by their children’s achievements yet paradoxically displayed a strong inclination to control their children’s lives and accomplishments. Maternal-Obligation mothers internalized maternal ideology, dedicating themselves to their children and interpreting their children’s success as a reflection of their own evaluation. However, they simultaneously also exhibited an accepting attitude toward the uncontrollable nature of their children’s successes and failures.

Mothers’ self-worth, influenced by their children’s academic outcomes, affects their parenting behaviors, which in turn impacts the emotional well-being of the child. In this sense, the parent–child relationship can be understood as interdependent (Seol et al., 2015). Type 1, which reflects a clear distinction between children’s achievements and the mother’s self-worth, demonstrates the lowest level of interdependence. In contrast, Type 2 and Type 4, characterized by higher levels of child-based self-worth and controlling parenting tendencies, indicate a high degree of parent–child interdependence. Type 3 appears to exhibit a mixed pattern of both separation and fusion.

### Limitations

This study had limitations that can provide directions for future research.

First, this study could not entirely exclude the possibility that negative perceptions of mothers who rely on their children’s achievements influenced the findings. In Korean society, children’s success is closely tied to parental self-worth, making this phenomenon challenging to objectively recognize and accept. Future research should employ in-depth qualitative methods (e.g., interviews, case studies) to explore mothers’ intrinsic motivations and emotional responses more thoroughly.

Second, although this study included mothers with children aged 1 to 20 years to account for differences based on children’s age, the sample was not evenly distributed across age groups, limiting the diversity of the findings. In addition, the sample was skewed toward employed mothers, which may have limited the representation of diverse occupational groups. Another limitation of this study is the lack of the sufficient collection of sociodemographic information, such as participants’ educational background, marital status, and regional characteristics. Future research should collect empirical data from a more diverse sample, considering a broader range of ages, socioeconomic backgrounds, and cultural differences, to enhance the generalizability of the results.

Third, this study focused exclusively on mothers as primary caregivers. However, with the increasing diversity of family structures in modern society, fathers, grandparents, and other guardians are increasingly taking on primary caregiving roles. Future research should expand the scope of participants to include various types of primary caregivers and conduct comparative studies to examine differences among them.

Fourth, this study did not require participants to consider a specific child during the Q sorting process. Consequently, in families with multiple children, maternal parenting attitudes and self-worth may vary depending on birth order or the child’s gender. Future research should take these variables into account to conduct a more refined and precise analysis.

Fifth, while the types identified in this study can serve as a foundation for counseling interventions, this study did not directly validate the effectiveness of tailored counseling and intervention strategies for each type. Future research should experimentally examine how type-specific counseling interventions impact parent–child relationships and mothers’ psychological well-being. Based on these findings, developing more refined and evidence-based parent counseling and education programs is essential.

Therefore, future research should address the limitations of this study by developing more refined assessment tools and empirically validating the effectiveness of type-specific counseling approaches. Such research could offer practical and evidence-based counseling and educational programs that meaningfully support clients.

## Figures and Tables

**Table 1:** Q methodology Procedure.

Steps	Procedures
Q concourse (population)	Literature research review Interviews
Q sample	Modification and improvement Repetitive comparative analysis n = 40
P sample	Considering the representativeness of the research objectives n = 33
Q sorting	Forced classification by P sample
Data analysis	Ken Q Analysis (2.0)

**Table 2:** Interviewee information.

No	Age	Years of Parenting	Number and Gender of Children	Employment Status
1	38	10 to less than 15 Years	2 Sons, 1 Daughter	Yes
2	40	5 to less than 10 Years	1 Daughter	No
3	45	15 to less than 20 Years	2 Daughters	Yes
4	47	15 to less than 20 Years	1 Son, 1 Daughter	No
5	50	15 to less than 20 Years	2 Daughters	Yes

**Table 3:** P samples and factor loading of each of the four types.

Types	No.	Factor Loading	Age	Years of Parenting	Number and Gender of Children	Job Category
**Type 1** n = 13	P28	0.8657	37	5 to less than 10 Years	1 Daughter	Employee
	P16	0.8245	45	10 to less than 15 Years	2 Daughters	Employee
	P8	0.79	37	5 to less than 10 Years	2 Daughters	Employee
	P6	0.699	39	5 to less than 10 Years	1 Son, 2 Daughters	Others
	P12	0.685	46	15 to less than 20 Years	1 Son, 2 Daughters	Employee
	P23	0.6577	37	5 to less than 10 Years	1 Daughter	Employee
	P25	0.6478	49	20 or more Years	1 Son, 1 Daughter	Employee
	P14	0.6441	42	5 to less than 10 Years	1 Daughter	Others
	P18	0.6368	39	5 to less than 10 Years	1 Son	Employee
	P22	0.6116	51	20 or more Years	2 Sons	Freelancer
	P3	0.5193	39	1 to less than 5 Years	1 Son	Homemaker
	P15	0.4965	48	15 to less than 20 Years	1 Son, 1 Daughter	Employee
	P10	−0.8458	50	15 to less than 20 Years	1 Daughter	Employee
**Type 2** n = 5	P7	0.858	52	15 to less than 20 Years	1 Son, 1 Daughter	Employee
	P13	0.6945	51	15 to less than 20 Years	1 Son, 1 Daughter	Employee
	P5	0.6557	49	15 to less than 20 Years	1 Son	Homemaker
	P4	0.6138	36	10 to less than 15 Years	1 Son, 1 Daughter	Self-employed
	P9	0.2532	52	15 to less than 20 Years	1 Son, 1 Daughter	Employee
**Type 3** n = 7	P27	0.8339	48	15 to less than 20 Years	1 Daughter	Employee
	P20	0.7026	47	15 to less than 20 Years	2 Sons	Employee
	P2	0.6917	47	15 to less than 20 Years	1 Son, 1 Daughter	Homemaker
	P24	0.6682	37	5 to less than 10 Years	1 Son	Employee
	P32	0.5998	39	10 to less than 15 Years	1 Son, 1 Daughter	Homemaker
	P26	0.5355	49	20 or more Years	1 Son, 1 Daughter	Employee
	P21	0.5095	44	1 to less than 5 Years	1 Daughter	Freelancer
**Type 4** n = 8	P19	0.6379	37	5 to less than 10 Years	1 Son	Employee
	P30	0.6374	41	10 to less than 15 Years	1 Son, 1 Daughter	Freelancer
	P33	0.6344	52	20 or more Years	1 Son	Freelancer
	P11	0.6278	45	10 to less than 15 Years	2 Daughters	Freelancer
	P29	0.6175	51	20 or more Years	1 Son, 1 Daughter	Employee
	P1	0.614	45	15 to less than 20 Years	2 Daughters	Freelancer
	P31	0.4821	47	10 to less than 15 Years	2 Daughters	Employee
	P17	0.3816	47	15 to less than 20 Years	1 Son, 1 Daughter	Self-employed

**Table 4:** Eigenvalues and explanatory variables of the four types.

Content/Type	Type 1	Type 2	Type 3	Type 4
Eigenvalues	11.54707049	3.208655	2.3764363	2.1214572
Explained Variance (%)	35	10	7	6
Cumulative Explained Variance (%)	35	45	52	58

**Table 5:** Q statement and Z-score by type.

Statement/Category	Type 1		Type 2		Type 3		Type 4	
	Z-Score	Q Sort Value	Z-Score	Q Sort Value	Z-Score	Q Sort Value	Z-Score	Q Sort Value
1. A child’s achievements serve as a measure of my success./**Fused**	−0.26	0	−2.16	−5 *	−0.34	−1	0.01	0
2. A child’s achievements reflect how well I have fulfilled my role as a mother./**Motherhood**	−0.28	−1 *	−1.39	−4 *	0.81	2	1.11	3
3. I can only gain recognition when my child achieves more./**Achievement**	−0.58	−1	−1.25	−4 *	−0.27	−1	0.1	0
4. My child is like an extension of myself, representing who I am./**Fused**	−0.29	−1	−1.36	−4 *	−0.1	0	0.55	1
5. I spare no expense or effort in ensuring my child’s success./**Fused**	0.54	1	−0.64	−1 *	1.56	5 *	0.85	2
6. I am satisfied with my life regardless of my child’s achievements or failures./**Independent**	1.36	4	−0.5	−1	1.4	4	−1.11	−3
7. My social status improves when my child achieves success./**Evaluation**	−0.12	0	−0.75	−2	−0.91	−3	−0.66	−2
8. When my child accomplishes something significant, my confidence in my own abilities increases./**Achievement**	0.03	1	−0.68	−1	−0.17	0	−0.89	−2
9. When my child fails, I tend to discipline them harshly./**Controlling**	−0.96	−2	−0.96	−3	−2.12	−5	−1.76	−5
10. I find it difficult to focus on my work because I worry about my child’s potential failures./**Fused**	−1.1	−4	−1.11	−3	−0.48	−1	−0.36	−1
11. My child’s achievements are a crucial factor in my mental health./**Fused**	0.01	1	−0.55	−1	1.27	4	1.38	4
12. How others perceive me is not influenced by my child’s achievements./**Independent**	1.28	3 *	−1.42	−5	0.08	1 *	−1.24	−4
13. When my child achieves success, I think of myself as a great mother./**Motherhood**	0.2	0 *	−0.41	−2	−1.09	−3	0.63	2 *
14. The most important goal in my life is to ensure my child’s success./**Achievement**	−0.47	−1	−0.9	−2	0.63	1	0.18	1
15. When my child achieves great success, I experience a sense of stability./**Evaluation**	0.68	2	0.67	1	0.3	1	1.2	3
16. My child’s achievements define my identity and prove who I am./**Achievement**	−0.62	−2	−0.72	−2	−1.36	−4	−0.44	−1
17. My child’s success or failure does not dictate the course of my life./**Independent**	1.95	5 *	−0.55	−1	−0.08	0	0.91	2 *
18. A mother’s sacrifice is necessary for her child’s success./**Motherhood**	0.62	2	0.36	0	1.11	3	1.24	3
19. I believe that both my child’s successes and failures are my responsibility./**Motherhood**	−1.07	−3	0.47	1 *	−0.35	−1 *	−1.31	−4
20. I set ambitious goals for my child to achieve success./**Achievement**	−0.05	0	0.23	0	−0.96	−3	−1.15	−3
21. When my child succeeds, I view myself in a more positive light./**Evaluation**	0.63	2	0.88	2	0.02	0	0.58	1
22 I want to do whatever it takes to prevent my child from experiencing failure./**Controlling**	−0.96	−2	0.93	3	1.16	3	−0.69	−2
23. When my child achieves success, it feels like a personal reward in my life./**Evaluation**	0.26	1	0.57	1	0.43	1	1.48	4 *
24. When my child achieves success, I grow to love myself more./**Achievement**	−0.54	−1	−1.06	−3	−1.31	−4	−0.45	−1
25. I wish I could take on my child’s failures for them./**Fused**	−0.98	−2 *	1.25	3	1.12	3	−0.02	0 *
26. When my child fails, I feel resentment toward them./**Controlling**	−1.77	−5	−0.41	0 *	−2.05	−5	−1.78	−5
27. I feel a sense of relief when my child achieves success./**Controlling**	0.6	1	1.26	4	0.89	2	1.54	5
28. I experience self-worth when I accomplish what I have planned, independent of my child./**Independent**	1.61	4	1.23	3	1.38	4	1.53	4
29. Even if my child achieves great success, my sense of self-worth remains unchanged./**Independent**	1.72	4	−0.17	0	1.87	5	0.13	1
30 I experience a sense of self-actualization through my child’s achievements./**Fused**	−0.07	0 *	1.45	4 *	−1.34	−4	−0.98	−3
31. When my child fails, I feel a deep sense of guilt and responsibility./**Evaluation**	−0.99	−3	0.85	2	−0.7	−2	0.61	1
32. When my child achieves success, I feel that my efforts have not been in vain./**Controlling**	1.03	3	1.53	5	−0.11	0 *	1.08	2
33. My child’s achievements serve as a source of new motivation for me./**Evaluation**	0.89	2	1.34	4	−0.66	−2	−0.03	0
34. I believe my child’s success is the result of my efforts./**Fused**	−1.22	−4	0.01	0	−0.55	−1	−0.72	−2
35. To prevent my child from failing, I take control over many aspects of their life./**Controlling**	−0.07	0	0.69	1	0.33	1	−0.33	0
36. When my child fails, the sense of satisfaction in life diminishes./**Independent**	−1.5	−5 *	0.45	1 *	−0.84	−2	−0.6	−1
37. The value of my personal life does not fully align with the value of being a mother./**Motherhood**	1.14	3	0.15	0	1.04	2	−0.52	−1
38. Academic success is the top priority in my child’s education./**Achievement**	−1.28	−4	0.85	2 *	−0.05	0 *	−1.64	−4
39. I believe my child’s failures are a natural part of their growth./**Controlling**	1.97	5	0.71	2	0.9	2	1.61	5
40. My child’s achievements are among the proudest moments of my life./**Achievement**	−1.05	−3	1.72	5 *	−0.58	−2	−0.05	0

* *p* < 0.05.

## Data Availability

The data are available from the corresponding author upon reasonable request.
